# Effectiveness of Group Physical Exercise in Treating Major Depressive Disorder: An Analysis of Secondary Data from an Aborted Randomized Trial

**DOI:** 10.3390/bs14030219

**Published:** 2024-03-08

**Authors:** Hossam Elgendy, Reham Shalaby, Belinda Agyapong, Deanna Lesage, Lacey Paulsen, Amy Delday, Sherianna Duiker, Shireen Surood, Yifeng Wei, Nnamdi Nkire, Vincent Israel Opoku Agyapong

**Affiliations:** 1 Department of Psychiatry, Faculty of Medicine and Dentistry, University of Alberta, Edmonton, AB T6G 2R3, Canada; hossamel@ualberta.ca (H.E.);; 2 Addiction and Mental Health, Alberta Health Services, Edmonton, AB T5J 0G5, Canada; 3 Department of Psychiatry, Faculty of Medicine, Dalhousie University, Halifax, NS B3H 4R2, Canada

**Keywords:** depression, exercise, physical activity, mental health, psychiatric patients

## Abstract

Background: Depression is highly prevalent and a significant cause of mortality and morbidity across the globe. Although antidepressants and/or psychotherapy are often used to treat depression, some recent studies indicate that exercise may play an important role in lowering depression symptoms among patients meeting the clinical criteria of a depressive episode. Objectives: This study aimed to evaluate the mental health and wellbeing of adult participants diagnosed with major depressive disorder (MDD) after fourteen weeks of receiving a supervised physical exercise program. Methods: In a pre-post design, the assessments were performed at baseline, seven weeks and fourteen weeks following the exercise intervention program using facilitated self-reported psychometric scales. The Beck Depression Inventory (BDI-2) and Clinical Outcomes in Routine Evaluation—Outcome Measure (CORE-OM) were used to assess depression. The short form of the International Physical Activity Questionnaire (IPAQ) was used for the self-reporting of participants’ physical activity. Results: At the beginning of the study, the baseline total mean scores and standard deviations for the BDI-2, CORE-OM, and IPAQ in both genders did not differ significantly (*p*-value > 0.05). Patients showed significant improvement in all assessment scales after completing fourteen weeks of the physical exercise program. Based on the BDI-2, the baseline score dropped from 31.25 (indicating moderate to severe depression) to 14.25 (indicating mild to minimal depression), with a *p*-value of <0.001. The CORE-OM total mean score was reduced from 1.91 to 0.98 with a significant *p*-value < 0.005 indicating effective clinical improvement in depression symptoms. The IPAQ total MET-minutes/week mean score increased from 1713.22 to 4367.62, indicating an improvement in the participants’ weekly P.E. intake; however, the change was not statistically significant *(p* = 0.07). Conclusions: Exercise treatment is linked with considerable therapeutic improvement in patients with MDD, particularly when exercise is sustained over time. The BDI-2 and CORE-OM total scores significantly decreased after the fourteen-week P.E. programme, indicating a change from moderate and severe depression to minimal and mild depression. Our findings offer insightful information to primary care doctors and psychiatrists, indicating that prescribing exercise to depressed patients may be a helpful adjunctive therapy.

## 1. Introduction

Depression is a mental disorder that affects millions of people globally every year. The global prevalence of depressive disorders is over 4%, and depression is the single largest contributor to nonfatal health loss [1]. The disease is characterized by chronic sorrow, despair, and a lack of interest in once-enjoyable activities [2]. It may significantly impact an individual’s quality of life and has many negative medical and psychological effects. There is a need to identify relatively cost-efficient, scalable interventions that can be offered to many people. Psychotherapy and pharmacotherapy have been the main lines of depression treatments. However, recently, some studies have shown that exercise may play an important role in reducing depression symptoms [2,3,4,5]. The magnitude of the effect of exercise as a treatment for depression is comparable to the magnitude of the effect of conventional treatment [1]. Antidepressant medications and exercise are thought to have complementary effects, synergistic benefits, and greater improvements in depressive symptoms compared to those who rely solely on medication [6,7]. It is important to note that while exercise can be a valuable adjunct to treatment, there is no clear evidence that exercise can be considered a substitute for professional medical treatment [7,8]. Individualized treatment plans are common for MDD patients, as different people may react differently to a given set of therapies. When creating a treatment plan, medical professionals consider variables including the patient’s preferences, the degree of their depression, and any contraindications [9,10]. A meta-analysis adjusting for publication bias concluded that “exercise has a large and significant antidepressant effect in people with depression” [3,11].

Physical exercise (any organized, controlled, and repeated bodily action to enhance or maintain one’s fitness and health) has several physiological and psychological advantages. An increasing amount of data shows that regular exercise may greatly help control and cure depression [5,12,13,14,15]. Physical exercise can improve mental function and reduce stress and depression. Aerobic exercise (cardiovascular exercise), such as running, has increased mood and mental wellbeing, reduced the risk of chronic illnesses, and improved cardiovascular health. Cycling is a low-impact workout that is gentle on the joints and may increase cardiovascular fitness and leg strength [16]. Resistance exercises such as weightlifting or using functional exercise with your body weight, such as push-ups and squatting, are another type of physical exercise that helps enhance metabolic rate, bone density, and muscular mass and promotes relaxation [17]. Exercises for flexibility and mobility, such as yoga and Pilates, can improve balance, flexibility, and relaxation.

Additionally, it could improve mental health by lowering stress and anxiety [18,19]. Body–mind exercises, such as Tai Chi, that encourage flexibility, relaxation, and balance, have been linked to improved physical and mental health [20]. Group exercise classes such as Zumba (A dance-based fitness class) incorporate aerobic activity, music, and dance techniques as a fun approach to raising cardiovascular fitness levels and enhancing mental condition. Women with fibromyalgia who practice Zumba may have less pain and have more functional ability [21]. Aquatic exercise is another physical exercise tool that has been demonstrated to reduce depression and anxiety and decrease oxidative stress in depressed elderly individuals [22,23].

There is a complex link between physical activity and depression and its underlying causes. Physical exercise affects neurobiology, endocrinology, and psychological wellbeing to boost mental health and emotional resilience [24,25].

Exercise releases mood-regulating neurotransmitters and neurotrophic substances, known as “feel-good” chemicals, that affect brain neurobiology. Those substances or chemicals are called endorphins; they are known to reduce pain and induce bliss. Exercise also boosts the synthesis of brain-derived neurotrophic factor (BDNF), stimulating neurogenesis, neuronal development, and synaptic connection. The mood and cognitive benefits for depressed patients may be due to exercise’s neurobiological effects [24,25,26,27,28,29]. Physical activity affects neurotransmitters and the endocrine system, particularly the hypothalamic–pituitary–adrenal (HPA) axis, which regulates stress responses. Regular exercise lowers cortisol levels and normalizes HPA axis function. These hormonal changes help offset persistent stress, a major risk factor for depression [24,28,29,30,31,32].

Moreover, regular physical exercise improves body image, self-esteem, and self-efficacy. Exercise may help people with depression feel in control of their lives. Group exercise and team sports may also provide a sense of belonging and social support, reducing depression sufferers’ feelings of isolation and loneliness [28,33]. According to the distraction hypothesis, exercising diverts the mind from anxieties and depressive thoughts. Distracting oneself from activities has generally been demonstrated to have a more favourable impact on managing depression and reducing depression than other coping mechanisms [2,34].

According to the thermogenic theory, the decrease in depressive symptoms is caused by increased core body temperature after exercise. The theory indicates that a general sense of relaxation and decreased muscle tension can result from temperature rises in certain brain areas, such as the brain stem. The study on the thermogenic hypothesis has focused on the impact of exercise solely on anxiety levels, not depression, even though elevated body temperature has been suggested as a mechanism for the association between exercise and depression [35,36,37]. More studies are required to determine which processes discussed here are significant modifiers of the impact of exercise. Biological, psychological, and social variables may affect how exercise and depression are related. This is in line with the way depression is now treated, which involves treating the patient’s biological, psychological, and social needs while addressing depression through the additive effects of medication and psychotherapy. Individual differences in the processes or systems mediating this link might exist [38]. This experimental study is an analysis of secondary data from an aborted randomized trial (due to the COVD-19 pandemic) that aimed to determine the feasibility and effectiveness of group exercise and cognitive behavioural therapy on depressive symptom scores and functioning among patients with depression [1]. Due to the limited capacity and resources secondary to the impact of COVID-19, this study focused on the generated data obtained from the exercise group. This research made use of every piece of information that could be obtained from the exercise regimen. The detailed methodology and secondary data analysis provided an extensive exploration of this research. The specific objectives included:

- To assess the effects of group physical exercise on depression symptoms and functional scores on standardized rating scales for patients with MDD;
- To assess the effects of a group physical exercise program on the self-reported physical activity of patients with MDD.

It was hypothesized that the group exercise program would result in a 25% reduction in depression symptoms as measured by the BDI-2 and a 25% improvement in wellbeing as measured by the COR-OM.

## 2. Methods

The study design, timeline, and participant selection for the original study, (registration number NCT03731728), which sought to compare group exercise with group cognitive behavioural therapy (CBT) for the treatment of MDD, are detailed in the published study protocol [1]. Due to restrictions brought on by the COVID-19 pandemic and technical challenges with organizing the group CBT program, the study was truncated mid-way, and this paper reports on secondary data from participants completing the group exercise program. Thus, this study is a one-arm, non-randomized experimental study with pre–post design of a secondary set of data of the original study. Participants in this study were recruited to receive 14 weeks of exercise intervention. The group exercise intervention was conducted in a recreational centre in Edmonton, Alberta, a large, socio-demographically diverse city in western Canada [1]. Patients who are assessed by a psychiatrist and diagnosed with a major depressive disorder (MDD) as per the *Diagnostic and Statistical Manual of Mental Disorders*, *Fifth Edition* (DSM-5) criteria for MDD [39,40] at the Edmonton Access 24/7 Addiction and Mental Health Clinic were offered an information leaflet inviting them to sign up for the study. Demographic characteristics were gathered at baseline. The names and contact details of the participants were gathered solely for future correspondence or scheduling treatment, evaluation, and follow-up appointments.

### 2.1. Selection Criteria

Inclusion criteria:

Age: between 18 and 65 years, MDD is the primary diagnosis ascertained by a psychiatrist or medical resident based on the criteria of the *Diagnostic and Statistical Manual for Mental Disorders, Fifth Edition* (DSM-5) [41].

Exclusion criteria:

Age: patients younger than 18 years or older than 65, with medical contraindications to exercise, such as cardiopulmonary disease preventing regular exercise or significant orthopedic problems. Primary psychiatric diagnosis other than MDD (e.g., psychosis, bipolar disorder, or schizoaffective disorder).

### 2.2. Interventions

Details of the exercise intervention are outlined in the original study protocol [1]. In brief, scheduled group exercises incorporated the following parameters: Aerobic or strength training exercises three times per week (60 min per session or 180 min per week) for 14 weeks. Moderate intensity exercise (participant’s self-rated physical activity of a 6 or 7 on the Borg Perceived Exertion Scale of 10 relative to the individual’s capacity). Moderate heart rate level was calculated (65–75% of maximum heart rate) for each participant at the beginning of the study [42,43]. All participants received instructions on how to take the heart rate manually. Fitness Alberta Leadership Certification Association-certified recreational therapists led the exercise sessions. Before the start of the study, the 2018 Physical Activity Readiness Questionnaire (PAR-Q+) was used to evaluate the safety of the patients’ participation in physical activity, and any physical issues were addressed to reduce the possibility of unfavorable events occurring during the sessions. PAR-Q+A is a screening tool for determining safe exercise participation before enrolling [40,44,45]. Participants deemed at risk for injury from physical activity were required to receive clearance from their medical doctor to participate before enrollment.

A variety of physical activities were offered to participants. Participants were empowered to decide on the activities (which make them meaningful). For a period of 14 weeks, participants could choose to participate in three of the following fitness regimens each week: Monday: individual fitness for 60 min; Tuesday and Thursday were circuit training/group exercise classes on the track—walk to a station, complete, then walk to next station, complete, etc., as a group or patients could do their fitness program in the gym area. Wednesday: aquafit or swimming for 60 min; two recreation therapists offered the participants programming for every session. The recreation therapists educated the participants about fitness training, health, and wellness, and how much effort goes into different types of exercise. Participants were advised to engage in activities at a moderate perceived exertion level (6–7 on a 10-point scale) for optimal outcomes. The participants used a rating of the perceived exertion scale to self-report their level of intensity to the facilitators while participating in the exercise programs [46,47].

### 2.3. Sample Size

Given that the original randomized trial was terminated due to COVID-19 pandemic restrictions, this study utilized all the data that could be gathered from the exercise program before the onset of the pandemic. Thus, the sample size for this single-arm study was not determined a priori.

### 2.4. Outcome Measures

Measures of depression, physical health, and mental wellbeing were taken at designated time points, including baseline, seven weeks (midpoint), and 14 weeks (exit) using the following validated psychometric instruments:

#### 2.4.1. BDI-2

The Beck Depression Inventory, second edition, is referred to as BDI-2. It is a popular self-report questionnaire used to assess the severity of depressive symptoms in individuals aged 13 years and older. The BDI-2 has 21 measures that measure different depressive symptoms, such as sorrow, lack of interest, guilt, and suicidal thoughts. Every item is scored from 0 to 3, with higher scores indicating greater depression symptoms. The BDI-2 has a total score range of 0 to 63, with higher values indicating more severe depression. To evaluate and track the progression of depression symptoms over time, it is frequently employed in clinical practice and research. The BDI-2 is scored by adding the scores for each of the 21 items. Each item is rated on a scale from 0 to 3. The scoring of this scale is measured as 0–13 indicates minimal depression; 14–19 indicates mild depression; 20–28 indicates moderate depression; 29–63 indicates severe depression [48,49,50,51].

Reliability: The BDI-II has demonstrated high internal consistency, suggesting that the scale’s components are assessing the same underlying construct for depression. For the BDI-II, Cronbach’s alpha coefficient, a popular indicator of internal consistency, is usually high. Cronbach’s alpha findings should provide a number between 0 and 1, although negative values are also possible. As a general rule, a Cronbach’s alpha of 0.70 and above is considered good, 0.80 and above is considered to be better, and 0.90 and above is considered to be the best [51,52]. In this study Cronbach’s alpha was calculated to assess the internal consistency reliability of the scale in a sample of 14 participants. The obtained alpha value was 0.76 indicating good internal consistency. This suggests that the items in the scale are homogeneous and measure a consistent underlying construct. Additionally, test–retest reliability refers to the consistency of scores over time. The BDI-II has demonstrated good test–retest reliability, suggesting that individuals tend to receive similar scores when retested under stable conditions [51,53]. The Shapiro–Wilk test of normality showed a significance of 0.256 (>0.05) with failure to reject null hypothesis and indicating that data follows normal distribution.

Validity—Content Validity: Clinical observations and depression diagnostic criteria were the foundation for developing the BDI-II. To guarantee that the scale sufficiently captures the spectrum of depressed symptoms, professionals in psychology and psychiatry participated in the item selection process. Additionally, studies have examined the BDI-II’s relationship with other measures of depression, demonstrating strong concurrent validity. It correlates well with other established measures of depression, supporting its validity as a tool for assessing depression [51,54].

#### 2.4.2. CORE-OM

Clinical Outcomes in Routine Evaluation—Outcome Measure (CORE-OM) is a common self-report questionnaire used to assess mental health and the psychological wellbeing of counselling clients. This metric records treatment outcomes and assesses mental health interventions. CORE-OM consists of 34 items, and respondents rate each one on a 5-point Likert scale ranging from 0 (Not at all) to 4 (Most of the time) [55,56], measuring a different aspect of mental health. Four psychological discomfort dimensions are included in the questionnaire, including:

- Wellbeing (4 items): This category investigates an individual’s positive feelings, life satisfaction, and wellbeing.
- Problems (12 items): It assesses the person’s psychological and emotional issues.
- Functioning (12 items): This domain assesses an individual’s professional, social, and relationship competencies.
- Risk (6 items): It investigates risk factors and challenges such as self-harm and suicidal ideation. CORE-OM has been validated and used in general care, mental health services, and counselling clinics.

Clinicians and researchers benefit from its comprehensive assessment of an individual’s emotional and psychological wellbeing. Some versions of CORE-OM also provide a total score, with some items reverse-coded. The total score represents an overall measure of an individual’s psychological wellbeing. Greater questionnaire scores imply greater psychological discomfort, whereas lower scores indicate better mental health [55,56,57]. The CORE-OM psychometric properties showed strong internal and test–retest reliability. All domains showed Cronbach’s α of >0.75 and <0.95 (i.e., appropriate internal reliability) [55,58,59]. The scale has strong convergent validity compared to a battery of current measures and physician risk assessments, as well as good sensitivity to modification [55]. Shapiro–Wilk test of normality showed a significance of 0.120 (>0.05) with failure to reject null hypothesis and indicating that data follows normal distribution.

#### 2.4.3. The International Physical Activity Questionnaire (IPAQ)

This is a widely used self-report instrument for determining an individual’s level of physical activity. An international team of researchers developed it and is widely used in epidemiological studies to gain additional insight into an individual’s physical activity patterns. The questionnaire assesses the frequency, duration, and intensity of physical activity, among other factors [60,61]. There are several versions of the IPAQ. The IPAQ Short Form was used in this study and is commonly used in lengthy surveys and research projects as it is easy to fill out. The amount of time spent walking and doing moderate-to-intense physical activity is the main concern [60,62]. Total physical activity is one of the primary outcomes of the IPAQ. It is typically expressed in the metabolic equivalent of task (MET)-minutes per week. The MET is the unit used to calculate the amount of energy used in different activities. The results of the IPAQ may be interpreted using the physical activity guidelines offered by health organisations. For instance, people should perform at least 150 min of moderate-intensity aerobic exercise or 75 min of vigorous-intensity aerobic activity each week, according to the World Health Organisation (WHO) [63].

**Continuous Scores on the IPAQ**

Median values and interquartile ranges can be computed for walking (W), moderate-intensity activities (M), vigorous-intensity activities (V) and a combined total physical activity score. All continuous scores are expressed in MET-minutes/week as defined below [60,62].

**MET Values and Formula for Computation of MET-minutes/week:**

An average MET score was calculated for every kind of exercise. For instance, a MET value for walking was developed by including all forms of walking. For both vigorous and moderately intense activities, the same protocol was followed. The IPAQ data analysis still makes use of the following values:

Walking = 3.3 METs, Moderate PA = 4.0 METs and Vigorous PA = 8.0 METs. Using these values, four continuous scores are defined:

Walking MET-minutes/week = 3.3 × walking minutes × walking days.

Moderate MET-minutes/week = 4.0 × moderate-intensity activity minutes × moderate days.

Vigorous MET-minutes/week = 8.0 × vigorous-intensity activity minutes × vigorous-intensity days.

Total physical activity MET-minutes/week = sum of Walking + Moderate + Vigorous MET-minutes/week scores [60,62,63,64,65,66].

**Categorical Score on the IPAQ**

**Category 1: Low** This is the lowest level of physical activity. Those individuals who do not meet the criteria for Categories 2 or 3 are considered to have a ‘low’ physical activity level [60,62].

**Category 2: Moderate** The pattern of activity to be classified as ‘moderate’ is either of the following criteria: Three or more days of vigorous-intensity activity of at least 20 min per day or five or more days of moderate-intensity activity and/or walking of at least 30 min per day; or five or more days of any combination of walking, moderate-intensity or vigorous intensity activities achieving a minimum total physical activity of at least 600 MET-minutes/week [60,62].

**Category 3: High** A separate category labelled ‘high’ can be computed to describe higher levels of participation. The two criteria for classification as ‘high’ are vigorous-intensity activity on at least three days, achieving a minimum total physical activity of at least 1500 MET-minutes/week, or seven days of any combination of walking, moderate-intensity or vigorous-intensity activities achieving a minimum total physical activity of at least 3000 MET-minutes/week [60,62].

**Sitting Question in IPAQ Short Form**

The IPAQ sitting question is an additional indicator variable of time spent in sedentary activity and is not included in any summary score of physical activity. The interquartile range and median values should be used when reporting data on sitting. Few studies have been conducted on sedentary (sitting) behaviours to date, and there are no established cut-off points for data that are displayed as categorical levels [60,62]. Regarding the reliability estimate (Cronbach’s alpha), the questionnaire was tested, and the results of the standardized Cronbach’s alpha test showed values between 0.63 to 0.85, being considered acceptable for the scale construction [67].

### 2.5. Follow-Up Assessment

Participants in the study completed the assessment tools at baseline, seven weeks after starting the program, and at the end of the study (14 weeks after the exercise commencement). These self-rated assessments were coordinated by a research facilitator who contacted the study participants and assisted them in completing a range of assessment tools related to the outcome measures.

### 2.6. Statistical Methods

Data were analyzed using SPSS for Windows version 25 (IBM Corporation) [68]. Descriptive data of sociodemographic characteristics, exercise-related, and clinical information were calculated and presented using frequency and percentage. The difference between the mean scores of BDI-2, CORE-OM, and IPAQ scales at baseline and 14 weeks following the administration of the exercise program was assessed using paired samples *t*-test. This test compares the means of two measurements collected from the same person, item, or unit of measurement. These “paired” measures might represent a measurement performed at two separate times, for example, a pre-test and post-test score with an intervention provided between the two time periods [69,70,71]. The scoring of this scale is measured as follows: 0–13 indicates minimal depression; 14–19 indicates mild depression; 20–28 indicates moderate depression; 29–63 indicates severe depression, and we examined the preference of depression using this category applying the chi-square test. We used the last observation carried forward (LOCF) method for imputing missing outcome data [72]. A standard methodology in many clinical fields for imputing incomplete longitudinal data sets is the (LOCF) method; the missing outcome is replaced by the last observed value (7th-week observations to replace 14th-week observations when they are missing). Missing data are particularly evident in mental health trials where dropout rates may exceed 50%, and the LOCF method is commonly applied [72,73,74,75]. The numbers reported represent the total responses recorded for each variable.

## 3. Results

Table 1 demonstrates the distribution of the study group’s sociodemographic and clinical variables. From the table, the majority of the participants were above 25 years old (10, 71.4%), had postsecondary education (8, 57.1%), and were employed (11, 78.6%). Concerning the exercise data, there were no significant differences in choice of types of offered exercise in both genders, with *p*-value > 0.05. About the clinical characteristics at baseline, there were no significant differences in the BDI-2, CORE-OM, and IPAQ baseline total mean scores and standard deviation in both genders at the beginning of the study, with *p*-value > 0.05. A total of 14 subscribers (6 males and 8 females) participated in the study, providing fourteen baseline responses to measurement scales. Out of the 14 subscribers, there were 6 dropouts, 8 participants (4 males and 4 females) completed the 7-week follow-up scales, and 5 participants completed the full 14 weeks of the program.

### Severity Analysis

Table 2 summarizes the difference in mean scores of the BDI-2, CORE-OM, and IPAQ, respectively, at the baseline and after fourteen weeks from the introduction of the exercise program. Out of the fourteen subscribers (five with moderate MDD and nine with severe MDD, based on the baseline BDI-2 score), eight participants (four with moderate MDD and four with severe MDD, based on the baseline BDI-2 score), half of them males and half females, have completed baseline and follow-up scales over the study time. The table shows significant changes in the mean scores of almost all scales pre- and post-exercise intervention. For BDI-2, the baseline score decreased from 31.25 (moderate to severe depression) to 14.25 (minimal to mild depression) with a *p* value of 0.001. The CORE-OM total mean score was reduced from 1.91 to 0.98 with a *p* value of 0.005, and the CORE-OM total mean score minus risk items was also reduced from 2.25 down to 1.14 with a *p* value of 0.005.

Furthermore, the CORE-OM mean score of risk items declined from 0.38 to 0.05 with a *p* value of 0.021. IPAQ total MET-minutes/week mean score was increased from 1713.22 to 4367.62, suggesting an improvement in the weekly amount of P.E. of the participants. However, it was not a significant change (*p* = 0.07).

All patients who completed the program had reported moderate to severe depression at baseline as measured by BDI-2. Six of them (75%) have shown improvement in depression symptoms, while two of them (25%) remained unchanged.

During the program, patients did not report any adverse effects from exercise, such as weariness, fatigue, tightness, or discomfort. Every participant in the intervention was able to perform the exercise without any issues.

**Figure 1:**
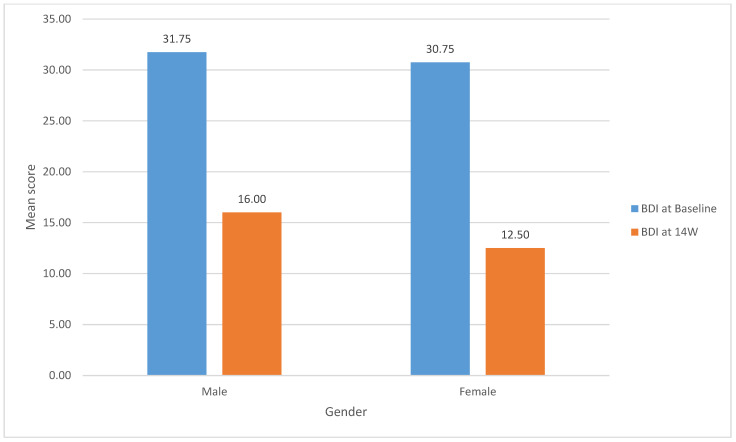
Change in the mean scores of BDI-2 by gender from baseline to fourteen weeks.

Figure 1 shows, among males, the BDI mean scores significantly dropped from (31.75, SD 6.24) at baseline to (16.00, SD 6.22) at 14 weeks.

(t (3) = 3.91, *p* = 0.03). Similarly, the females’ BDI mean scores significantly dropped from (30.75, SD 7.63) at baseline to (12.50, SD 10.72) at 14 weeks (t (3) = 5.66, *p* = 0.01).

## 4. Discussion

This study was a clinical trial that used a pre–post design that examined the effectiveness of physical exercise (P.E.) on improving major depression symptoms and wellbeing in a group of male and female participants who were diagnosed with MDD symptoms. All the participants were on antidepressant medication during the study period. Various physical exercise activities were offered to the study participants, such as aquafit, group exercise, independent cardio, and individual fitness. There was no statistically significant gender difference regarding the choice or preference of a specific type of P.E. Our results suggest that physical exercise helped support mental health and wellbeing and significantly improve depressive symptoms among the study participants. At the end of the fourteen-week P.E. program, there was a significant reduction in the severity of depression as measured by the mean scores of the BDI-2 scale (from 31.25 representing moderate to severe depression at baseline to 14.25 representing minimal to mild depression) at 14 weeks, with high effect size (Cohen’s d: 2.47).

Similarly, the CORE-OM total score decreased from 65.75 to 33.25 with a significant *p* value < 0.004. The IPAQ short form of the total MET mean score showed improvement in the participants’ physical activity from 1713.22 prior to intervention up to 4367.62 after the intervention; however, the results were not statistically insignificant, rather approaching significance (*p* = 0.070). This observation might result from the study’s small sample size, which suggests the study was underpowered to illicit a significant improvement in physical activity levels among study participants. Our findings are consistent with the outcome of a study carried out by Imboden et al., 2020, which found significant improvement in depression symptoms after receiving a daily low-intensity aerobic exercise (A.E.) program for six weeks [76], as measured by the Hamilton Depression Rating Scale (HRSD) [77,78]. Using a different measuring scale in this study and obtaining the same results provides significant support to the reliability of the scales we used. Buschert et al. found similar effects of A.E. in a depressed patient sample (*n* = 38) [79], and Netz, Y., reported an equally positive effect of A.E. versus treatment as usual (TAU) [8]. P.E. has also proved to positively impact other life aspects of patients who live with depression. Schuch, F.B. et al. and Kerling et al. reported a significant effect of P.E. as an add-on tool to the usual treatment in terms of improved quality of life among patients with severe depression, using the Brief Generic Quality of Life Assessment scale (WHOQOL BREF) [80,81]. Likewise, Craft et al., in their review, reported that the exercise programs *reduced* overall symptoms of depression and were more effective than other lines of treatment [2]. Knubben et al. reported significant antidepressant effects of short-term exercise for ten days on the mood of patients with major depression assessed using the Bech–Rafaelsen Melancholy scale (BRMS) [71] and the Center for Epidemiologic Studies Depression Scale (CES-D) [82].

Antidepressant medications were administered to all our patients; the antidepressants include various classes of medications, such as selective serotonin reuptake inhibitors (SSRIs), serotonin-norepinephrine reuptake inhibitors (SNRIs), and others. They work by affecting neurotransmitter levels in the brain, particularly serotonin and/or norepinephrine [83]. Many studies have reported that there is a significant positive impact on the treatment of MDD patients when physical exercise is combined with conventional antidepressant medications [76,84,85,86]. Combining these two approaches is known as an integrative or multimodal treatment strategy [87]. Nahas, R et al. and Xie Y et al. found that this positive effect may be attributed to a complementary effect, where it is believed that exercise and antidepressant drugs work best together. Neurotransmitter levels in the brain may be regulated by antidepressants, and exercise has been demonstrated to improve mood and reduce stress [6,86]. Additionally, Guerrera, C.S. et al. suggested that combining antidepressants with regular exercise may have synergistic effects that enhance the course of depression therapy. Exercise can improve general mental health and may even amplify the benefits of antidepressant drugs [7]. Furthermore, Schuch, F.B. et al., and Ren, J. et al. found that exercise and pharmaceuticals have been demonstrated to affect neurobiological elements linked to depression, including increased neurogenesis (the formation of new neurons) and alterations in neurotransmitter levels. The combination could reduce depression symptoms in several ways [88,89].

### 4.1. Recommendations and Future Directives

Based on the study results, primary care physicians and mental health providers are encouraged to advise patients with depression to engage in physical exercise since there is mounting evidence to support the effectiveness of this behavioural intervention in lowering depression symptoms. However, a few pragmatic issues must be considered when recommending physical exercise as an adjunctive treatment. First, patients with depression tend to be sedentary and may lack the enthusiasm to start an exercise regimen. According to the American College of Sports Medicine [90], adults should engage in moderate-to-intense physical activity for at least 30 min daily, five days a week, or more. For patients with depression who are often inactive, such a recommendation is perhaps daunting. According to the meta-analytic results in this field, 20 min of moderate-intensity exercise three times a week is enough to significantly lessen symptoms of depression. As a result, healthcare providers may start with this regimen as an initial guideline [91]. Healthcare providers can advise patients to take it easy at first and select a pleasurable exercise style. Walking is typically a cost-effective choice for many individuals, and the physician should advise patients to choose a safe area to walk and a place to stroll inside during bad weather (e.g., a shopping mall). The primary care physician may wish to advise the patient to start by increasing the frequency of his or her exercise activities, for example, by establishing a target of three exercise sessions per week [2,92]. According to Martinsen and Berger et al., exercise intensity is an important aspect of exercise that must be considered. It is also good to remind patients that exercise does not have to be long or intensive to improve mood. Moderate-intensity exercise (60–80% maximum heart rate) is often more enjoyable than more vigorous exercise for individuals with low fitness levels, which is a characteristic of most depressed patients [92,93].

Furthermore, Sallis et al. reported that starting a moderate-intensity exercise program is linked to a dropout rate in the general population that is approximately half that of starting a vigorous-intensity exercise program [94]. Therefore, encouraging patients to start with lower-intensity, pleasant exercise should result in a more positive exercise experience for the patient and enhance the probability that they will continue to participate in exercise. Further studies are required to evaluate each type of P.E. on the treatment outcome of depressed patients.

### 4.2. Limitations

This research has limitations that must be addressed when evaluating the results. The low number of patients in the study is a limitation. The authors are aware that a small sample size may not adequately represent the broader population. Consequently, it becomes challenging to generalize the study findings to a larger group of individuals as well as inadequate capture of heterogeneity within the population. Individual differences and diverse characteristics may be overlooked, limiting the applicability of the findings. Additionally, small sample sizes may not be sensitive enough to detect small or subtle effects, even if they are clinically meaningful, as well as the possibility of potential bias whether due to participant selection, measurement error, or other confounding factors. The use of antidepressant medications alongside exercise was a source of possible bias; in fact, antidepressant medications were administered to all patients in our research. Therefore, it is still a concern that improvement might be due to pharmacotherapy or the combination therapy, rather than exercise alone, which is a further limitation. Another limitation is the lack of a control group that did not receive the P.E. intervention, which makes it harder to control for confounding variables that could influence the results. The absence of a control group makes it difficult to distinguish between the effects of the intervention and placebo effects. Participants may experience improvements simply due to their belief in the efficacy of the intervention. This can lead to biased or spurious associations between the intervention and outcomes. Furthermore, the positive effect of exercise was calculated collectively without comparing the difference in the effect of each type of exercise selected by the patients; this limitation could be attributed to the low number of participants.

## 5. Conclusions and Implications for Policy and Practice

The results of this clinical trial provide helpful information for administrators and clinicians interested in integrating exercise interventions into current MDD care. Our pre–post trial investigating the impact of P.E. on MDD symptoms in a diverse group of participants demonstrated significant improvements in mental health and a clinical reduction in depressive symptoms. The fourteen-week P.E. program led to a substantial decrease in BDI-2 and CORE-OM total scores, indicating a shift from moderate and severe depression to minimal and mild depression. While the IPAQ short form showed only a trend towards significant improvement in physical activity, the proximity to significance suggests a potential impact that a larger sample size might elucidate. Encouragingly, our results provide valuable insights for healthcare professionals, suggesting that advising patients with depression to engage in exercise can be a beneficial adjunctive treatment. However, pragmatic considerations, such as the passive nature of depressed individuals, should be acknowledged. Recommending a gradual approach with pleasurable, moderate-intensity exercises, like walking, appears to be a practical starting point. Our study supports the idea that even shorter and less intensive exercise sessions can significantly alleviate depressive symptoms. While this study underscores the positive impact of P.E. on depression, it acknowledges the need for further research to explore the effects of specific exercise types on treatment outcomes for patients with depression. In moving forward, these findings advocate for a nuanced approach to integrating physical exercise into depression management, recognizing its potential as an accessible, scalable, and effective intervention.

## Figures and Tables

**Table 1:** Distribution of socio-demographic, exercise-related, and clinical characteristics.

Variables	Male *n* = 6	Female *n* = 8	Total (%)	
**Age group**				
≤25 year	2 (33.3%)	2 (25%)	4 (28.6%)	
26–40 year	4 (66.7%)	1 (12.5%)	5 (35.7%)	
>40 year	0 (0.0%)	5 (62.5%)	5 (35.7%)	
**Education level**				
High school diploma or less	3 (50.0%)	1 (12.5%)	4 (28.6%)	
Postsecondary education	3 (50.0%)	5 (62.5%)	8 (57.1%)	
Student	0.0 (0.0%)	2 (25%)	2 (14.3)	
**Employment status**				
Employed	5 (83.0%)	6 (75.0%)	11 (78.6%)	
Unemployed	1 (16.7%)	2 (25.0%)	3 (21.4%)	
**Exercise Choices**				
Choice 1				
Individual fitness	5 (83.3%)	5 (62.5%)	10 (71.4%)	
Group exercise	1 (16.7%)	2 (25%)	3 (21.4%)	
Aquafit	0 (0.00%)	1 (12.5%)	1 (7.1%)	
Choice 2				
Group exercise	3 (50%)	1 (12.5%)	4 (28.6%)	
Aquafit	3 (50%)	6 (75%)	9 (64.3%)	
Independent cardio	0 (0.00%)	1 (12.5%)	1 (7.1%)	
Choice 3				
Hiking	1 (16.7%)	5 (62.5%)	6 (42.9%)	
Group exercise	4 (66.7%)	1 (12.5%)	5 (35.7%)	
Independent cardio	0 (0.00%)	2 (25%)	2 (14.3%)	
Walking	1 (16.7%)	0 (0.00%)	1 (7.1%)	
**Types of exercise, mean (S.D.)**			***t* value (pdf)**	***p*** **-value**
Aquafit	3.33 (3.72)	6.13 (4.42)	−1.24 (12)	0.723
Group exercise	11.83 (7.02)	11.25 (14.43)	0.091 (12)	0.075
Independent cardio	2.67 (4.54)	1.25 (2.37)	0.760 (12)	0.080
Individual fitness	4.67 (4.80)	5.00 (4.30)	−0.137 (12)	0.907
Walking	6.17 (5.91)	3.13 (3.22)	1.24 (12)	0.162
Total weekly activities	28.67 (18.57)	26.75 (22.01)	−172 (12)	0.374
**Measures baselines, mean (S.D.)**				
BDI-2 All score baseline	33.50 (6.09)	31.37 (6.84)	0.602 (12)	0.559
CORE-OM All score baseline	2.00 (0.58)	2.06 (0.27)	−0.263 (12)	0.217
IPAQ Baseline Total MET-minutes/week	2260.80 (4042.72)	1512 (1798.25)	0.470 (12)	0.647

**Table 2:** Changes in baseline mean scores of BDI-2, CORE-OM, and IPAQ scales after the introduction of the exercise program.

Measure	Scores			Change from Baseline, %	Mean Difference (95% CI)	*p* Value	*t* Value (df)	Effect Size (Cohen’s d)
	n	Baseline Score, Mean. (S.D.)	Fourteen-Week Score, Mean. (S.D.)					
BDI-2	8	31.25	14.25		17.00	<0.01	6.98	2.47
		(6.47)	(8.32)	54.40	(11.24–22.75)		(7)	
CORE-OM mean score total	8	1.91	0.98		0.93	<0.01	4.03	1.42
		(0.49)	(0.49)	48.69	(0.38–1.47)		(7)	
CORE-OM mean score risk items	8	0.38	0.05		0.33	0.02	2.97	1.05
		(0.39)	(0.11)	86.84	(0.06–0.59)		(7)	
CORE-OM total mean score minus risk items	8	2.25	1.14		1.11	<0.01	3.99	1.41
		(0.66)	(0.57)	49.33	(0.45–1.76)		(7)	
IPAQ total MET minutes/week mean score	8	1713.22	4367.62		−2654.40	0.07	−2.13	−0.76
		(3574.39)	(3729.90)	154.93	(−5592.03–283.23)		(7)	

## Data Availability

The data presented in this study are available on request from the corresponding author. The data are not publicly available due to privacy and ethical reasons.
